# Thyroid-Specific Genes Expression Uncovered Age-Related Differences in Pediatric Thyroid Carcinomas

**DOI:** 10.1155/2016/1956740

**Published:** 2016-02-28

**Authors:** Maria Isabel Cunha Vieira Cordioli, Lais Moraes, Maria Teresa de Seixas Alves, Rosana Delcelo, Osmar Monte, Carlos Alberto Longui, Adriano Namo Cury, Janete Maria Cerutti

**Affiliations:** ^1^Genetic Bases of Thyroid Tumors Laboratory, Division of Genetics, Department of Morphology and Genetics and Division of Endocrinology, Department of Medicine, Universidade Federal de São Paulo, Pedro de Toledo 669, 11 Andar, 04039-032 São Paulo, SP, Brazil; ^2^Pediatric Section, Department of Pathology, Universidade Federal de São Paulo, Rua Botucatu 740, 04023-900 São Paulo, SP, Brazil; ^3^Thyroid Section, Department of Pathology, Universidade Federal de São Paulo, Rua Botucatu 740, 04023-900 São Paulo, SP, Brazil; ^4^Molecular Medicine Laboratory, Irmandade da Santa Casa de Misericórdia de São Paulo, Rua Dr. Cesário Mota Jr. 112, 01221-020 São Paulo, SP, Brazil; ^5^Division of Endocrinology, Irmandade da Santa Casa de Misericórdia de São Paulo, Rua Dr. Cesário Mota Jr. 112, 01221-020 São Paulo, SP, Brazil

## Abstract

Despite a more advanced stage of disease at presentation, a better response to radioiodine (RAI) therapy and a reduced overall mortality have been reported in pediatric differentiated thyroid cancer (DTC) in comparison to adult DTC. Few studies suggested that the better response to RAI therapy in pediatric patients might be associated with an increased expression of NIS. However, a marked heterogeneity within the pediatric group has been recognized. Children (<10 years old) usually present a more aggressive disease than adolescents (≥10–18 years old). By analyzing the expression of thyroid-specific genes in 38 sporadic pediatric tumors, we show that the expression of NIS, PDS, and TSHR was lower in children than adolescents (*P* < 0.05). A linear regression confirmed the association between NIS expression and age. Most significantly, NIS was expressed at similar levels in DTC from children and adults, whereas PDS and TSHR expression was even lower in DTC from children, compared to adolescents and adults. Our data suggest that biological behaviors of DTC in adolescents might differ from those in children and adults. Therefore, the premise that the expression of thyroid-specific genes is higher in tumors from pediatric patients than in adults is not entirely true and might be too oversimplified.

## 1. Introduction

Thyroid cancer is the fastest increasing cancer worldwide [1]. Although the highest incidence rates are observed in the fifth decade and it is rare in the younger population, the incidence of thyroid cancer is also increasing in children (<10 years old) and adolescents (≥10–18 years old) [2, 3]. Thyroid cancer is the 2nd most prevalent cancer in females aged 15 to 19 years [4]. Similar to adults (>18 years old), differentiated thyroid carcinomas (DTC) are the most common malignancy, with nearly 75–90% being papillary thyroid carcinoma (PTC) and the remainder follicular thyroid carcinoma (FTC) [2].

Previous studies reported significant differences in the clinical presentation and outcomes of DTC in pediatric patients (≤18 years old) compared to adults [5, 6]. Despite a more advanced stage of disease at presentation and higher rates of recurrences than adults, the overall mortality is lower [7–9]. Unlike adults, pediatric patients have a higher prevalence of pulmonary metastases, which almost always are functional [10–12]. This may explain why pediatric patients with DTC have better responsiveness to radioiodine (RAI) therapy than the adults [10, 11]. In fact, some studies have shown that most pediatric DTC patients had a complete remission after RAI therapy, mainly those adolescents with iodine-avid pulmonary metastases [12–14].

The better response to RAI therapy in pediatric patients might infer greater degree of differentiation and higher expression of proteins involved in iodine uptake and metabolism. The transport of iodide into thyroid follicular cells is a result of an active transport mechanism mediated by the sodium iodide symporter (NIS) protein, an integral plasma membrane glycoprotein located at the basolateral membrane of thyroid follicular cells [15]. Following active transport across the membrane, iodide is translocated across the apical membrane by pendrin (PDS) and organified by thyroid peroxidase (TPO). These actions are reliant on the thyroid-stimulating hormone (TSH), which interacts with the TSH receptor (TSHR) at the basolateral membrane of follicular cells [15].

Therefore, there is a strong need to investigate whether the expression of proteins involved in iodine uptake and metabolism differs between pediatric and adult populations. In fact, it has been suggested that* NIS* expression was associated with lower risk of recurrence of pediatric thyroid carcinomas [16, 17]. Additionally, the dose of 131I required to achieve remission was directly related to the levels of* NIS* expression, being higher in those patients with undetectable* NIS* expression [17, 18]. The expression of* PDS* was also found diminished in pediatric patients [5].

Nevertheless, a marked heterogeneity within the pediatric group has been reported. Some earlier studies suggested that children present with more aggressive local disease and are more likely to have lymph node metastases at diagnosis and, probably, more prone to develop subsequent distant metastases than adolescents [2, 7, 8, 11, 19, 20]. It has also been reported that children experience recurrence more frequently and earlier than adolescents [21, 22].

Therefore, there is a strong need for studies in both children and adolescents. Although efforts have been made, the role of RAI therapy in pediatric patients has been mainly assessed in adolescents. Most studies used a very small number of children or prepubertal patients [10, 23], which makes it difficult to stratify pediatric patients into age groups or pubertal status and to test the hypothesis that the expression of genes associated with iodine uptake and metabolism might be higher in adolescents than in children. As most studies are retrospective or included a small number of children, it still remains unclear whether age influences the behavior of DTC within pediatric population [24].

To bridge some of the existing gap, this study investigated the expression of* NIS*,* PDS*,* TPO*,* TSHR*, and thyroglobulin (*TG*) in a cohort of pediatric patients and correlated with clinicopathological features. To further explore a possible association of the expression of thyroid-specific genes with age, the expression analysis was performed in pediatric patients stratified into two age groups (<10 and ≥10–18 years). As our analysis suggests an impact of age on gene expression, the genes whose expression pattern differed between children and adolescents were further compared with their expression in DTC from adults.

## 2. Materials and Methods

### 2.1. Thyroid Samples

The series consists of 47 formalin-fixed paraffin-embedded (FFPE) sections from 38 primary tumors from pediatric patients who underwent thyroid surgery at Hospital São Paulo (Universidade Federal de São Paulo) and Hospital da Santa Casa de São Paulo between the years 1993 and 2012. The pediatric cohort included 35 PTCs, 3 FTCs, and 9-matched normal thyroid tissues. All samples were reviewed by two pathologists (RD and MTSA). The clinical and pathological features are summarized in Table 1.

As recommended by the ATA guidelines for children with thyroid nodules and DTC, all of the pediatric patients were ≤18 years of age at the time of diagnosis [24]. The pediatric cases were further separated into two age groups: children (<10 years old) and adolescents (≥10–18 years old). Because information about pubertal development was not available for all of the patients, the age of 10 was used as the cut-off point. This cut-off point was recommended by the World Health Organization (WHO) and used to determine the effect of age on time to recurrence and mortality rates in pediatric DTC [21, 22].

The adult cohort included 115 PTC and 7 adjacent normal thyroid tissues obtained from patients who underwent thyroid surgery from 2000 through 2007 at Hospital São Paulo (Universidade Federal de São Paulo) and Hospital das Clínicas de São Paulo (Universidade de São Paulo). The clinical and pathological features are summarized in Table 2.

The control groups (normal thyroid tissues) included only those samples from the contralateral nodule of patients with unilateral DTC (i.e., no evidence of either benign or malignant thyroid disease in the contralateral nodule). The study was conducted under the approval of the Review Boards and Research Ethical Committees of the affiliated institutions.

### 2.2. RNA Isolation and cDNA Synthesis

Total RNA was isolated from 10 *μ*m thick FFPE sections using the Recover All Total Nucleic Acid isolation kit (Ambion Inc., Austin, TX). Total RNA (500 ng) was treated with DNAse and reverse-transcribed into cDNA with oligo-dT_12–18_ (50 *μ*M) and random hexamers (50 ng) using a Superscript III transcriptase kit (Invitrogen Corp., Carlsbad, CA).

### 2.3. Expression of Thyroid-Specific Genes in Thyroid Samples by Quantitative RT-PCR

The thyroid samples were screened for the expression of target genes (*NIS, TG, TPO, PDS,* and* TSHR*) and the reference gene (*RPS8*) by quantitative RT-PCR (qRT-PCR), as previously described [25, 26]. Briefly, an aliquot of cDNA was used in a 12 *μ*L PCR reaction containing SYBR Green PCR Master Mix (PE Applied Biosystems) and 3 pmol of each specific primer. The PCR reaction was subjected to 40 cycles at 95°C for 15 seconds and 60°C for 1 minute. The qPCR reactions were performed in triplicate, and the Ct was obtained and averaged (SD < 0.85). The relative expression (RE) was calculated according to the comparative ΔΔCt method. Normal thyroid samples were used as the control group. The choice to use the* RPS8* as the reference gene is based on previous analyses from our group that identified this gene as the best suitable reference gene for thyroid tissues [27]. The PCR primers are summarized in Supplementary Table 1 (see Supplementary Material available online at http://dx.doi.org/10.1155/2016/1956740).

### 2.4. Statistical Analysis

Statistical analysis was performed using GraphPad Prism 6.0 (GraphPad Software, La Jolla, CA, USA) and R 3.1.3. (R software). Mann-Whitney *U* test or Student's *t*-test was used to compare the continuous variables, and Fisher's exact test was used for dichotomous variables. The associations were tested using discriminant analysis or linear regression when applicable. The results with *P* < 0.05 were considered to be statistically significant.

## 3. Results

### 3.1. Clinical and Pathological Features of Pediatric Patients

The mean age at diagnosis was 11.84 years (range, 4 to 18 years). The median age of the pediatric control group was 11.7 years (range from 7 to 17 years) and, therefore, similar to that observed in pediatric thyroid cancer group. The female to male ratio was 29 : 9, with female predominance mainly in the adolescent group. The pathological findings included multifocality in 17 (45%), extrathyroidal extension in 16 (42%), lymph node metastases at diagnosis in 28 (74%), and lung metastasis in 10 (26%) patients. Two of the patients had a family history for PTC, and four cases had a history of previous radiation exposure during childhood to treat another cancer (Table 1). When compared to adults, higher rate of cervical metastases was identified in pediatric patients compared to adults (74% versus 42%; *P* = 0.0007), as well as pulmonary metastases (26% versus 3%; *P* < 0.0002) (Table 2).

To better understand clinical, pathological and expression differences between children and adolescents, the patients were classified into two age groups. Thirteen cases (34%) were <10 years of age, and 25 cases (66%) were ≥10–18 years of age. An increased prevalence of extrathyroidal extension was observed among the children compared to the adolescents (69% versus 28%; *P* = 0.0448). Children also had a higher tendency to develop lung metastasis than adolescents (46% versus 16%; *P* = 0.0620) (Table 1).

### 3.2. Expression of Thyroid-Specific Genes and Correlation with Clinical and Pathological Features and Molecular Status

**Figure 1:**
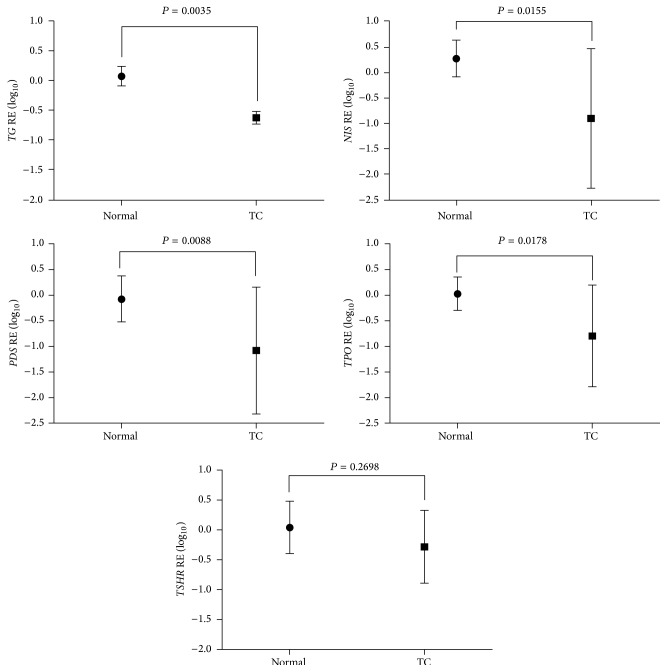
Relative expression (RE) of thyroid-specific genes in differentiated thyroid carcinomas (DTC) (*n* = 38) and normal thyroid tissues (*n* = 9) from pediatric patients. The graphics shows the mean value (±SD) of log-transformed data. *P* < 0.05 were considered statistically significant.

**Figure 2:**
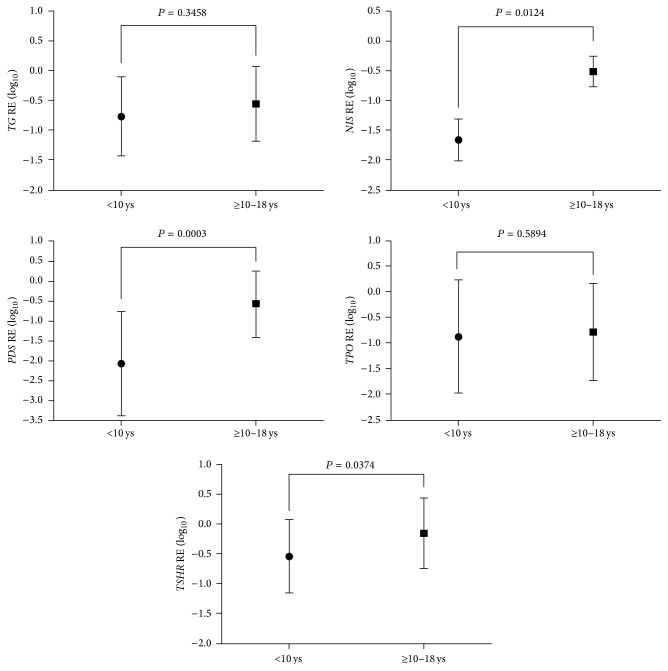
Relative expression (RE) of thyroid-specific genes in pediatric thyroid carcinomas classified according age: children (*n* = 13; <10 years old) or adolescents (*n* = 25; ≥10 years old). The graphics shows the mean (±SD). Data was log-transformed before analysis. *P* < 0.05 were considered statistically significant.

**Figure 3:**
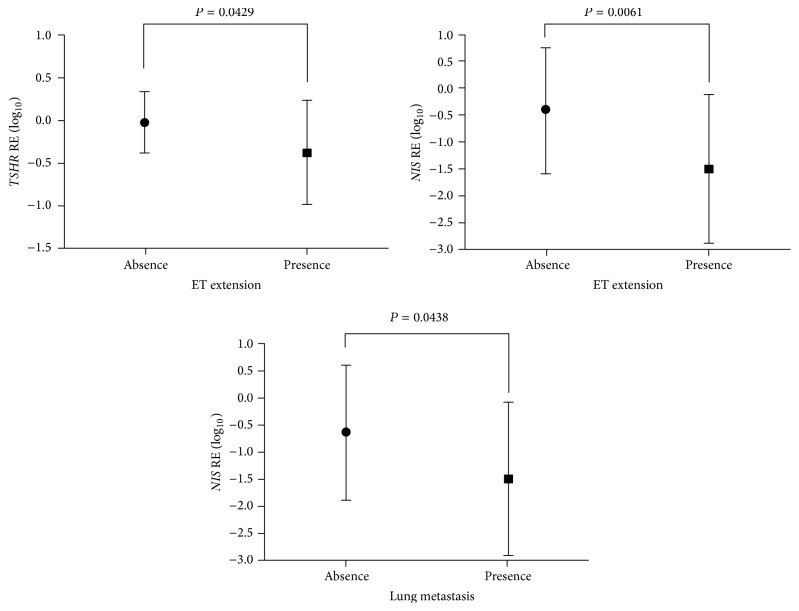
Relative expression (RE) of* TSHR* and* NIS* in pediatric thyroid carcinomas classified according to the presence (*n* = 22) or absence (*n* = 16) of extrathyroidal (ET) extension; and RE expression of* NIS* in patients classified according to the presence (*n* = 10) or absence (*n* = 28) of distant metastasis. The graphics shows the mean value (±SD) of log-transformed data. *P* < 0.05 were considered statistically significant.

The expression of* TG*,* NIS*,* PDS*, and* TPO* was significantly lower in pediatric thyroid carcinomas compared to the normal thyroid tissue (Figure 1; *P* < 0.05). Importantly, the expression of* NIS*,* PDS,* and* TSHR* was consistently lower in children than in adolescents (Figure 2; *P* < 0.05). Considering the clinical and pathological features associated with poor prognosis,* NIS* and* TSHR* mRNA expression was significantly lower in those tumors with extrathyroidal extension. Besides,* NIS* mRNA expression was notably lower in those tumors from patients with lung metastases (Figure 3). Multiple linear regression analysis confirmed the association between age and* NIS* expression, regardless of the other clinicopathological features.

**Figure 4:**
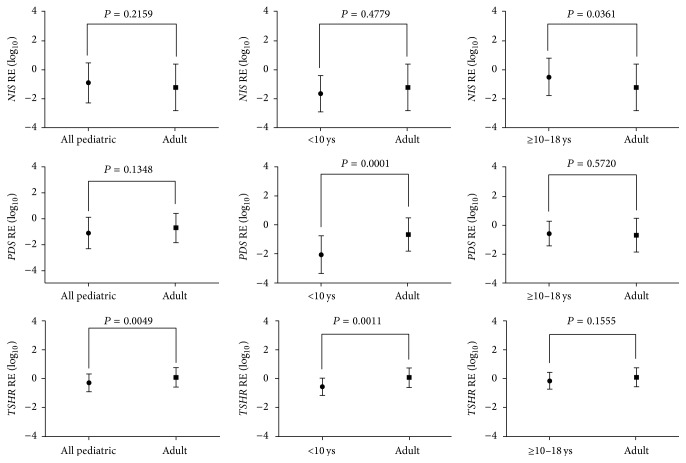
Relative expression (RE) of* NIS*,* PDS*, and* TSHR* in DTC patients stratified by age: all pediatric patients (≤18 years old; *n* = 38), children (<10 years old; *n* = 13), adolescents (≥10–18 years old; *n* = 25), and adults (≥19 years old; *n* = 115). The graphics shows the mean (±SD) expression level of log-transformed data. *P* < 0.05 were considered statistically significant.

As* NIS*,* PDS*, and* TSHR* were differentially expressed between children and adolescent groups, we further compared those changes to the changes we observed in adult population [25, 26]. We observed here that children and adults in have similar* NIS* mRNA expression. Remarkably,* NIS* expression was significantly higher in tumors from adolescents than tumors from adults (*P* < 0.05) (Figure 4). The range of* TSHR* and* PDS* expression in children was considerably lower as compared to adults (*P* < 0.05) (Figure 4).

Seeing that adult population is characterized by a wide age range (20–70 years) and that older patients have more aggressive disease, we separated younger adults (<45 year old) from older adults and evaluated the expression of* NIS, PDS,* and* TSHR* in these groups. However, the expression of these genes did not differ between these two age groups (data not presented).

## 4. Discussion

Previous studies reported significant differences in the clinical presentation and outcomes of DTC among pediatric patients when compared to adults. Pediatric patients have larger tumor size, have more extensive local disease, are more likely to present with lymph node and distant metastases, and have a higher frequency of functional metastases. The discrepancy between more aggressive disease at diagnosis and higher recurrence rates but a more favorable progression-free survival is quite remarkable [11]. It is still not clear whether these differences observed between pediatric and adult DTC lie in the existence of distinct gene expression and/or mutational profile. It has been suggested that the rare progression to less-differentiated tumor and the better response to RAI therapy are due to higher expression of key genes involved in thyroid function, including* NIS* [11].

Another intriguing point is the heterogeneity reported within pediatric group. As most studies included a very small number of children, it is still unclear whether younger age is associated with an increased risk for extensive disease. Studies, in which the number of children is roughly 25% of the pediatric cohort, showed that young age is correlated with a higher risk for extensive disease or recurrence [8, 19, 20].

In this work, we identified a significant decrease in* TG, NIS, PDS,* and* TPO* expression in pediatric DTC compared to paired-normal thyroid tissues. Moreover, the age-related analysis revealed that the expression of* NIS*,* PDS*, and* TSHR* was significantly lower in children than in adolescents. Remarkably, children had a higher rate of extrathyroidal extension and a trend towards a higher prevalence of distant metastases than adolescents.

Although one study previously investigated the expression of* NIS* in children and adolescents, no significant difference in* NIS* expression was found between benign and malignant thyroid tumors [17]. Though no difference was found between benign and malignant tumors, because the overall recurrence risk was increased for tumors that had undetectable* NIS* expression, the authors suggested that* NIS* expression is a favorable prognostic indicator for DTC in children and adolescents [17]. Although these results appear to be in contrast with ours, in the former study, the expression of* NIS* in the malignant tumor was compared to benign lesions as a substitute of normal thyroid. In the present study the expression of* NIS* in malignant tumors was compared to its expression in normal thyroid. That is essential, as previous studies have demonstrated that* NIS* expression was lower in benign thyroid lesion compared to normal thyroid tissue [28, 29]. Additionally, the authors studied patients up to 21 years of age and only 2 cases under the age of 10. Hence, no comparison was made between children and adolescents. Remarkably,* NIS* expression was not detected in these patients under the age of 10 [17].

Another study suggested that the expression of* PDS*,* TPO*, and* TSHR* mRNA is higher in the pediatric group (5–21 years) compared to adults (22–59 years). Nevertheless, among the 15 pediatric patients, only 3 cases were under 10 years of age, and there was no specific information regarding the expression of iodine-metabolizing genes in these patients [16].

As in our study the expression of* NIS*,* PDS*, and* TSHR* was significantly different between children and adolescents; the premise that the expression of iodine-metabolizing genes is higher in all DTC from pediatric patients than in DTC from adults might not be entirely true and seems too oversimplified. The differences observed among children and adolescents in the present study, at molecular level, may explain the striking differences reported within the pediatric group in terms of the clinical and pathological features.

To help to clarify the issue whether the level of expression of iodide-metabolizing genes in children and adolescents differs from that of adults, we next compared the expression of* NIS*,* PDS*, and* TSHR*, which were found differentially expressed between children and adolescents, with their expression in adult population. We found that the expression of* NIS* is comparable in tumors from children and adults. Most significantly, the expression of* NIS* was higher in tumor from adolescents compared with children and adults.* PDS* and* TSHR* expression were even lower in children than in adolescents and adults. The higher expression of* NIS, PDS,* and* TSHR* in adolescents suggested a greater degree of differentiation of thyroid carcinomas in this age group. The opposite is also true, that is, lower expression of this thyroid-specific genes may indicate a lower grade of tumor differentiation and, therefore, a more aggressive thyroid tumor behavior in children.

There are some limitations to our study. First, it is limited by the inherent biases of a retrospective analysis and therefore the lack of a proper follow-up of the patients. Second, because previously published guidelines about DTC management were mainly addressed for adult DTC, the management of pediatric DTC varies according to different medical services. The patients enrolled in this study were followed in three different hospitals with different criteria regarding the surgical procedures, indications for RAI ablation and dosing regimens, and follow-up protocol. The first guideline specifically addressing the management of thyroid nodules and DTC in children and adolescents was only recently released [24]. For this reason, we decided not to analyze the relationship between gene expression and response to RAI treatment.

In conclusion, to the best of our knowledge, this is the first study to report a differential thyroid-specific gene expression profile within the pediatric group classified according to age. Our data suggests that the biological behavior of tumors in adolescents is different compared to tumors in patients under the age of 10 and adults. The identification of age-related differences may allow a subclassification of pediatric tumors into genetic and clinical subtypes and will certainly add to our ability to predict clinical outcomes and to develop future treatment strategies tailored to the differences. Whether genetic events might explain the phenotypic differences warrants further investigation.

## Supplementary Material

The supplementary table provides the information about PCR expected product size and the primers sequences for the thyroid-specific genes (NIS, PDS, TG, TPO and TSH-R), and for the reference gene (RPS8).

## Figures and Tables

**Table 1:** Summary of the clinicopathological features of pediatric thyroid carcinoma.

	Total	Patients <10 yr old	Patients ≥10–18 yr old	*P* value
	*n* = 38	*n* = 13	*n* = 25	
Mean age ± SD	11.84 (±4.4)	6.76 (±1.92)	14.48 (±2.63)	<0.0001
Gender				
Female	29	8 (62%)	21 (84%)	0.2262
Male	9	5 (38%)	3 (16%)	
Tumor size (cm) mean ± SD	2.65 (±1.48)	2.28 (±1.49)	2.83 (±1.48)	0.2383
Risk factors				
Family history	2	1 (8%)	1 (4%)	1.00
Exposure to radiation	4	0	4 (16%)	0.2779
Extrathyroidal extension	16	9 (69%)	7 (28%)	**0.0448**
Multifocal disease	17	4 (31%)	13 (52%)	0.3068
LN metastases	28	11 (85%)	17 (68%)	0.4413
Distant metastases	10	6 (46%)	4 (16%)	0.0620

**Table 2:** Summary of the clinicopathological features of pediatric and adult thyroid carcinoma.

	Pediatric	Adult	*P* value
	*n* = 38	*n* = 115	
Mean age ± SD	11.84 (4–18 ys)	45.29 (20–76)	
Gender			
Female	29 (76%)	96 (83%)	0.3387
Male	9 (24%)	19 (17%)	
Extrathyroidal extension	16 (42%)	39 (34%)	0.5596
Multifocal disease	17 (45%)	56 (49%)	0.7082
LN metastases	28 (74%)	48 (42%)	**0.0007**
Distant metastases	10 (26%)	4 (3%)	**0.0002**
