# Supplementary material for: Thyroid-Specific Genes Expression Uncovered Age-Related Differences in Pediatric Thyroid Carcinomas
Source: Int J Endocrinol. 2016 Feb 28;2016:1956740. doi: 10.1155/2016/1956740 (PMC4789067; doi:10.1155/2016/1956740)
Supplement: Supplementary file 1 — The supplementary table provides the information about PCR expected product size and the primers sequences for the thyroid-specific genes (NIS, PDS, TG, TPO and TSH-R), and for the reference gene (RPS8). [file 1956740.f1.doc]

**Supplementary Table 1.** Primer sequences and expected PCR product size

| **Genes** |  | **Primer Sequence**  **(5' - 3')** | **Expected Product size (bp)** |
| --- | --- | --- | --- |
|  |  |  |  |
| ***TSHR^a^*** | Sense | ACATGACGTCAATCCCTGTG | 105 |
|  | Antisense | TGAAAGCATATCCTTGGACTG |  |
| ***NIS^a^*** | Sense | CAGAACCACTCCCGGATCAA | 81 |
|  | Antisense | ACCCACCACAAAAGTCCAGAA |  |
| ***TPO*** | Sense | TTGTACAACGGGTTCCCACT | 103 |
|  | Antisense | GGAGGTCAGAATAGCGGTCA |  |
| ***PDS*** | Sense | TCAAGAGGGTCAAGGTTCCA | 102 |
|  | Antisense | TCAAGTTCTTCTTCCGTCAGC |  |
| ***TG*** | Sense | TTCAGTGAGCTGCTCCCCAATC | 165 |
|  | Antisense | ATCTTCTCTTAGCCCAGATCCAGCC |  |
| ***S8^a^*** | Sense | AACAAGAAATACCGTGCCC | 125 |
|  | Antisense | GTACGAACCAGCTCGTTATTA |  |

a [27]
